# Perioperative, function, and positive surgical margin in extraperitoneal versus transperitoneal single port robot-assisted radical prostatectomy: a systematic review and meta-analysis

**DOI:** 10.1186/s12957-023-03272-7

**Published:** 2023-12-12

**Authors:** Yu Jiang, Yang Liu, Shize Qin, Shuting Zhong, Xiaohua Huang

**Affiliations:** 1 Department of Radiology, Affiliated Hospital of North Sichuan Medical College, Nanchong, China; 2 Department of Urology, Affiliated Hospital of North Sichuan Medical College, Nanchong, China

**Keywords:** Prostate cancer, Single port, Robot-assisted radical prostatectomy, Extraperitoneal approach, Transabdominal approach, Meta-analysis

## Abstract

**Background:**

Extraperitoneal and transperitoneal approaches are two common modalities in single-port (SP) robot-assisted radical prostatectomy (RARP), but differences in safety and efficacy between the two remain controversial. This study aimed to compare the perioperative, function, and positive surgical margin of extraperitoneal with transperitoneal approaches SP-RARP.

**Methods:**

Following the Preferred Reporting Items for Systematic Reviews and Meta-Analyses (PRISMA) statement, this study is registered with PROSPERO (CRD 42023409667). We systematically searched databases including PubMed, Embase, Web of Science, and Cochrane Library to identify relevant studies published up to February 2023. Stata 15.1 software was used to analyze and calculate the risk ratio (RR) and weighted mean difference (WMD).

**Results:**

A total of five studies, including 833 participants, were included in this study. The SP-TPRP group is superior to the SP-EPRP group in intraoperative blood loss (WMD: − 43.92, 95% CI − 69.81, − 18.04; *p* = 0.001), the incidence of postoperative Clavien-Dindo grade II and above complications (RR: 0.55, 95% CI − 0.31, 0.99; *p* = 0.04), and postoperative continence recovery (RR: 1.23, 95% CI 1.05, 1.45; *p* = 0.04). Conversely, the hospitalization stays (WMD: 7.88, 95% confidence interval: 0.65, 15.1; *p* = 0.03) for the SP-EPRP group was shorter than that of the SP-TPRP group. However, there was no significant difference in operation time, postoperative pain score, total incidence of postoperative complications, and positive surgical margin (PSM) rates between the two groups (*p* > 0.05).

**Conclusions:**

This study demonstrates that both extraperitoneal and extraperitoneal SP-RARP approaches are safe and effective. SP-TPRP is superior to SP-EPRP in postoperative blood loss, the incidence of postoperative Clavien-Dindo grade II and above complications, and postoperative continence recovery, but it is accompanied by longer hospital stays.

**Supplementary Information:**

The online version contains supplementary material available at 10.1186/s12957-023-03272-7.

## Introduction

Minimally invasive surgery has gained widespread popularity globally, and robot-assisted radical prostatectomy (RARP) has emerged as a prominent surgical approach for localized prostate cancer treatment [1–3]. In recent years, with the rise of extraperitoneal radical prostatectomy (EPRP), it has become a common surgical approach like transperitoneal radical prostatectomy (TPRP) [4, 5]. Some scholars advocate that EPRP can reduce operation time, blood loss, and postoperative hospital stay while reducing positive surgical margin (PSM) and the incidence of postoperative complications. Conversely, other scholars assert that TPRP provides an improved surgical field of vision, heightened surgical precision, and better safeguarding of the urethra and nerve tissue. In addition, many researchers compared the two surgical approaches. Previous meta-analyses have indicated that EPRP has faster operation time, shorter postoperative hospital stay, and lower postoperative complication rates compared with TPRP [6, 7].

The single-port (SP) RARP was approved by the USA for the treatment of radical prostatectomy in 2018. It has the characteristics of a compact body, minimal trauma, and dual cameras, and it has attracted more and more attention. The SP-RARP can reduce surgical invasiveness and complication rate and can be used as an alternative to traditional multiport RARP [8–10]. The SP-RARP can also achieve both extraperitoneal and transperitoneal surgical approaches, yet the disparities in safety and efficacy between these approaches remain unclear, leaving the debate over the optimal surgical technique unresolved. Therefore, it is necessary to summarize and analyze the current studies comparing the two surgical approaches of SP-RARP to fill in the gaps in this field.

This study endeavors to compare the perioperative, function, and positive surgical margin of extraperitoneal with transperitoneal approaches SP-RARP and provide valuable insights to clinicians in their selection of the most suitable surgical method.

## Methods

Our meta-analysis adheres to the Cochrane Preferred Reporting Items for Systematic Reviews and Meta-Analyses (PRISMA) guidelines [11] and was registered with PROSPERO (CRD42023409667) before the study started.

### Search strategy

Up to February 2023, we have searched the databases of PubMed, Embase, Web of Science, and Cochrane Library to compare the studies of SP-TPRP and SP-EPRP in the treatment of prostate cancer. We construct the keywords according to the principles of PICOS and use the combination of subject words and free words to search: (((((transperitoneal radical prostatectomy) OR (TPRP)) OR (extraperitoneal radical prostatectomy)) OR (EPRP)) AND (((Robotic Surgical Procedures) OR (Robotics)) OR (Robot-assisted))) AND ((SP) OR (single port)). Additionally, we manually retrieved and reviewed the relevant references of the papers to ensure comprehensiveness and minimize potential omissions.

### Study selection

We defined inclusion criteria according to PICOS principles. P(patients): Patients diagnosed with localized prostate cancer; I (intervention): the patient underwent extraperitoneal single-hole robot-assisted radical prostatectomy; C (comparator): the patient underwent SP-EPRP or SP TPRP; O (outcome): The included studies include one or more outcome indicators: perioperative outcome, functional outcome, and oncological outcome; S (study type): a case–control study, cohort study, or randomized controlled trial. Exclusion criteria: (1) conference reports, editorial comments, and conference abstracts; (2) non-comparative research; and (3) no data analysis available.

### Data collection

The two observers independently extracted the following data from the research we included (1) general information: first author, publication years, and country; (2) population characteristics: patient age, body mass index (BMI), prostate-specific antigen (PSA) level, prostate size, and tumor stage; (3) perioperative outcomes: operation time, hospital stay, intraoperative blood loss, and postoperative pain score; (4) postoperative total complications; (5) postoperative complications of II grade or above (defined as Clavien-Dindo grade ≥ II); (6) continence recovery (defined as the using no pad or one safety pad/day); and (7) positive surgical margins (PSM). Discrepancies were resolved by a third reviewer.

### Bias risk assessment

We used the Newcastle–Ottawa Scale (NOS) (https://www.ohri.ca//programs/clinical_epidemiology/oxford.asp) to evaluate the quality of included non-randomized controlled trials and excluded those with scores < 5, including bias due to (1) case selection, (2) comparability, and (3) outcome reporting. Two reviewers assessed the quality and evidence of the study and resolved the differences through discussion.

### Statistical analysis

A meta-analysis was carried out using the Stata15.1 software (StataSE, USA). The results of the dichotomous variables are reported by the risk ratio (RR) and its 95% confidence interval (CI), and the results of the continuous variables are reported by the weighted mean difference (WMD) and its 95%CI. Statistical heterogeneity was evaluated based on *I*^2^ statistics. The *I*^2^ ≥ 50% indicates significant heterogeneity, using a random effect model, and when *I*^2^ < 50%, a fixed effect model is used. We use the leave-one-out method for sensitivity analysis to ensure the stability of the results, that is, delete each study in turn and observe the changes in the results. However, we cannot conduct a sensitivity analysis of three or fewer studies. *P* ≤ 0.05 means the difference is statistically significant [12].

## Results

### Baseline characteristics

**Fig. 1:**
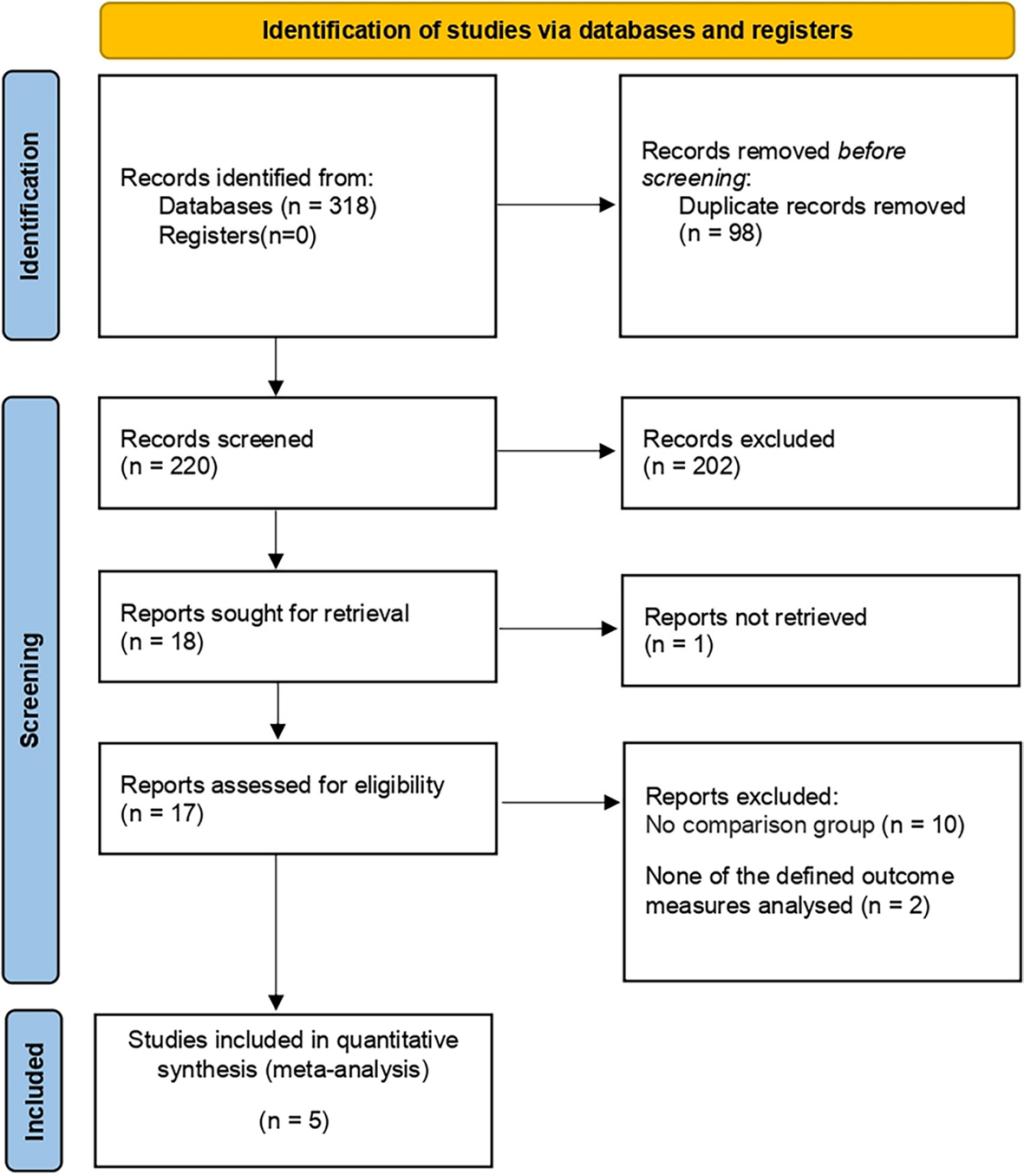
Literature screening flowchart

We searched 318 studies from 4 databases, excluded repeated studies, then screened them according to PICOS principles, and finally obtained five studies [10, 13–16]. The five studies (sample sizes ranging from 34 to 476) were prospective or retrospective cohort studies conducted in the USA, with a total of 833 patients (425 SP-TPRP and 408 SP-EPRP). For more information on the screening process, please see (Fig. 1).

Table 1 summarizes the baseline characteristics and preoperative variables (including prostate volume, pathological stage, age, body weight, etc.). In addition, the two groups were balanced in age (*p* = 0.957), BMI (*p* = 0.054), preoperative PSA (*p* = 0.139), previous abdominal surgery (*p* = 0.394), biopsy grade (*p* = 0.995), and prostate volume (*p* = 0.458) (Supplementary File 1). Table 2 summarizes the perioperative period, function, and positive surgical margin of various studies.

**Table 1:** Characteristics of included studies

Study	Abaza 2020 [13]		Kaouk 2020 [14]		Balasubramanian 2022 [16]		Zeinab 2022 [15]		Zeinab 2023 [10]	
Country	USA		USA		USA		USA		USA	
Operative approach	Transperitoneal	Extraperitoneal	Transperitoneal	Extraperitoneal	Transperitoneal	Extraperitoneal	Transperitoneal	Extraperitoneal	Transperitoneal	Extraperitoneal
Number of patients, *N*	24	10	46	52	39	30	78	78	238	238
Age, years	61.27(7.2)		61.1(6.9)	62.5(6.0)	62.7(6.8)	64.6(8.6)	61.5 (5.78)	62.5 (6.67)	63.0 (7.4)	64.0 (5.9)
Bia, kg/m^2^	27.1(4.3)		29.34(5.3)	29.5(4.9)	28.8(4.3)	32.1(6.4)	28.3 (4.3)	27.4 (4.3)	27.0 (3.7)	27.0 (3.7)
Spa, ng/ml	7.8(8.1)		10.6(8.5)	8.0(5.7)	7.4(5)	9.1(5.5)	5.9 (2.9)	5.5 (2.6)	6.5 (0.9)	6.5 (3)
Follow-up time	NA		NA	NA	150(214.1)	150(214.1)	7(3.7)	9(5.9)	6.0 (6.7)	7.0 (7.5)
Previous abdominal surgery, *N* (%)	0	0	15(33%)	12(23%)	6(15.4%)	6(20.0%)	37.0 (47.4%)	27.0 (34.6%)	1.0 (0.6%)	49.0 (28.5%)
Prostate volume, mL	NA		NA	NA	51.8(35.1)	48.3(17.4)	33.0 (12.2)	30.0 (15.3)	49.0 (12.6)	49.0 (14.8)
Pathologic stage, *N* (%)										
≤ pT2	NA		27 (60.0%)	27(51.9%)	21(53.8%)	16(53.3%)	NA		107.0 (72.3%)	111.0 (61.7%)
> pT2	NA		19 (41.3%)	25(48.1%)	12(30.8%)	8(26.7%)	NA		41(27.6%)	69(38.4%)
Biopsy grade group, *n* (%)										
GrGp1	NA		2 (4.3%)	2(3.8%)	NA		24.0 (31.2%)	15.0 (19.5%)	39.0 (16.4%)	47.0 (19.7%)
GrGp2			28 (60.9%)	33 (63.5%)			35.0 (45.5%)	50.0 (64.9%)	195.0(81.9%)	188.0(79.0%)
GrGp3			9 (19.6%)	8 (15.4%)			15.0 (19.5%)	10.0 (13.0%)		
GrGp4–5			7 (15.2%)	9 (17.3%)			3.0(3.9%)	2.0(2.6%)	4.0(1.7%)	3.0(1.3%)

^a^Biopsy Grade Group

^b^Biopsy Grade Group

^c^International Society of Urological Pathology (ISUP) grading of prostate cancer was used

**Table 2:** Perioperative outcome

Study	Abaza 2020 [13]		Kaouk 2020 [14]		Balasubramanian 2022 [16]		Zeinab 2022 [15]		Zeinab 2023 [10]	
Operative approach	Transperitoneal	Extraperitoneal	Transperitoneal	Extraperitoneal	Transperitoneal	Extraperitoneal	Transperitoneal	Extraperitoneal	Transperitoneal	Extraperitoneal
Lymphadenectomy, *N* (%)	0	0	24(52%)	51(98%)	37(94.9%)	23(76.7%)	35.0 (45.5%)	76.0 (97.4%)	101.0 (52.9%)	192.0 (84.6%)
Lymph nodes resected	0	0	12 (3.7)	5 (2.2)	4.9(3.4)	4.5(4.6)	4.0 (8.2)	5.0 (2.8)	9.0 (8.3)	6.0 (3.7)
Nerve-sparing (unilateral or bilateral), *N* (%)	NA		31(67.4%)	45(86.5%)	NA		56 (96.6%)	47 (81.0%)	NA	
Operative time, minutes	171.9 (26)	198.1 (34.8)	248.2(42.3)	201(37.5)	248(36)	224(41)	210.0 (37.1)	190.0 (28.2)	155.0(103.2)	206.0(42.6)
Estimated blood loss, mL	112.5 (53.1)	152.5 (82)	117.6(93.7)	145.7(87.7)	130(70)	138(87)	100.0 (74.1)	150.0 (74.1)	75.0 (74.2)	150.0 (74.2)
Hospital stays (days or hours)	NA		25.7 (14.4)	4.3 (10.7)	1.05(0.2)	1.1(0.4)	5.5 (13.3)	4.6 (8.4)	14.0 (8.1)	7.5 (14.7)
Catheterization time, days	NA		NA		NA		3.0 (0.7)	7.0 (0.7)	5.0 (0.9)	7.0 (2.2)
Pain	NA		Pain score at discharge 1 (2.9)	Pain score at discharge 2 (2.2)	Post-Operative Opioid Requirement (*N* = 23)	Post-Operative Opioid Requirement (*N* = 24)	Pain score at discharge 3.4 (1.9)	Pain score at discharge 3.7 (2.1)	Pain score at discharge 2.0 (0.7)	Pain score at discharge 2.0 (2.2)
PSM, *N* (%)	NA		19(41.3%)	14 (26.9%)	10(25.6%)	8(26.7%)	12.0 (15.4%)	20.0 (25.6%)	61.0 (26.9%)	55.0 (23.3%)
Continence, *N* (%)	NA		24(60%)	25(62.5%)	30(76.9%)	15(51.7%)	56 (96.6%)	47 (81.0%)	133.0 (63.0%)	87.0 (53.0%)
Postoperative complications, *N* (%)	NA									
Overall	NA		7(15.2%)	6(11.5%)	2(5.1%)	3(10%)	10.0 (12.8%)	11.0 (14.1%)	32.0 (13.4%)	39.0 (16.4%)
Clavien-Dindo grade ≤ 2	NA		4(8.6%)	2(3.8%)	NA	NA	9(11.5%)	6(7.7%)	22.0 (68.7%)	21.0 (53.8%)
Clavien-Dindo grade > 2	NA		3(6.5%)	4(7.7%)	2(5.1%)	3(10%)	2(2.5%)	5(6.4%)	10(31.2%)	18.0 (46.1%)
Complications type, *N* (%)										
Lymphocele	NA		1.0(2.2%)	3.0(5.8%)	0.0 (0.0%)	1.0(3.3%)	1.0 (1.3%)	6.0 (7.7%)	7.0 (21.9%)	14.0 (35.9%)
Pelvic abscess	NA		1.0(2.2%)	1.0(1.9%)	NA		1.0 (1.3%)	0.0 (0.0%)	NA	
Urinary tract infection	NA		3.0(6.5%)	1.0(1.9%)	NA		0.0 (0.0%)	1.0 (1.3%)	8.0 (25.0%)	2.0 (5.1%)
Others (ileus, bladder spasm, hematuria)	NA		1.0(2.2%)	2.0(3.8%)	NA		8(10.2%)	4.0(5.2%)	7.0(21.9%)	16.0(41%)

### Assessment of quality

The NOS scale was used to score the included literature. All studies had a score of ≥ 5 with a median of 7, with one study [16] scoring 7 and two studies [10, 15] scoring 8. See Table 3 for details.

**Table 3:** The risk of bias table

Study	Selection	Comparability	Outcome	Overall score
Abaza 2020 [13]	★★	★★	★	5
Balasubramanian 2022 [16]	★★★	★★	★★	7
Kaouk 2020 [14]	★★★	★★	★	6
Zeinab 2022 [15]	★★★	★★	★★★	8
Zeinab 2023 [10]	★★★	★★	★★★	8

### Outcome analysis

#### Perioperative outcomes

**Fig. 2:**
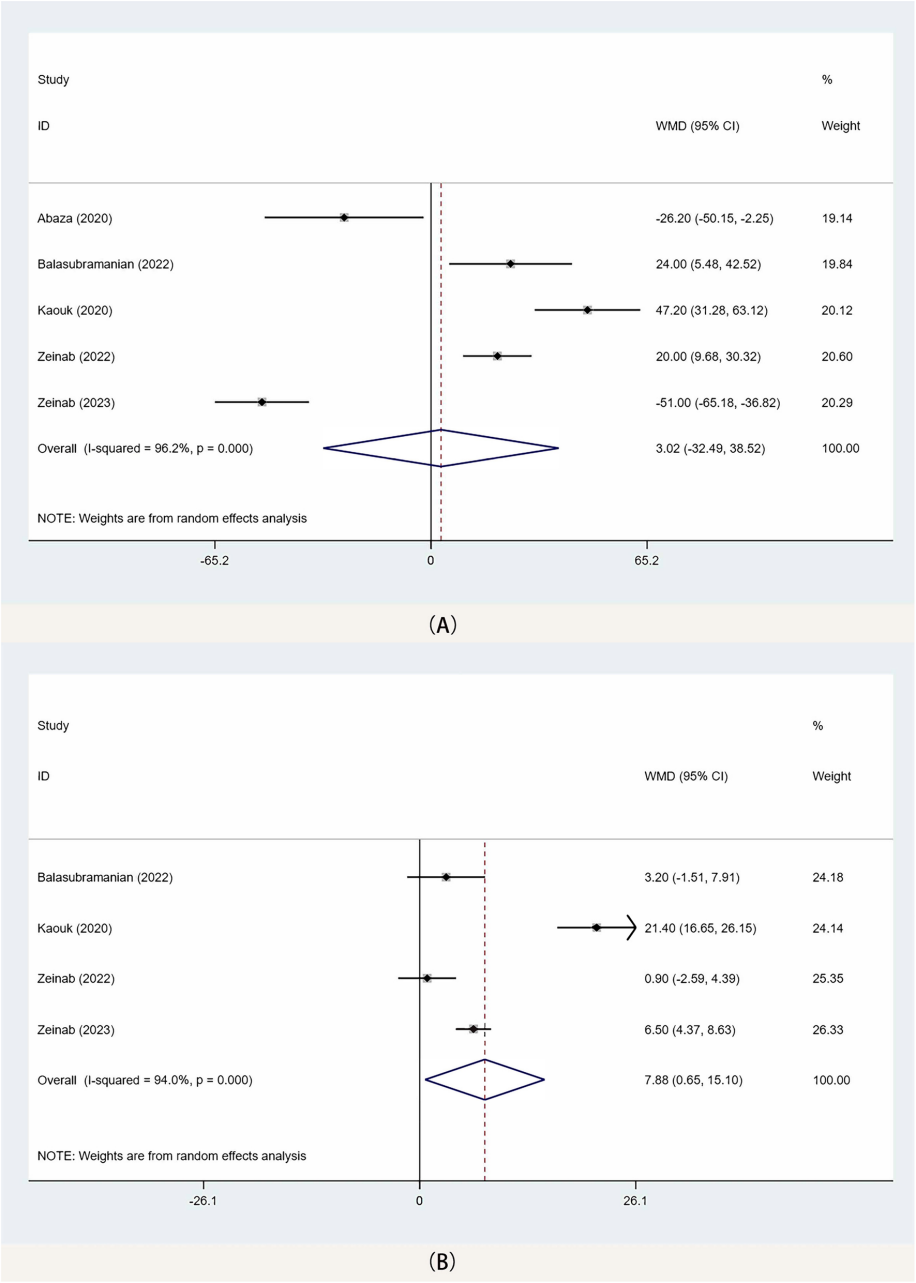
Forest plots of perioperative outcomes for SP-TPRP vs SP-EPRP. (**A** operative time, **B** hospital stay). WMD weighted mean difference, CI confidence interval

A total of 5 studies [10, 13–16] reported the operative time for both SP-EPRP and SP-TPRP surgical approaches, and the meta-analysis results showed no significant difference in operative time (WMD: 3.02 min, 95% CI − 32.49, 38.52; *p* = 0.868) between the two groups (Fig. 2A).

The meta-analysis of 4 studies [10, 14–16] showed that SP-TPRP was associated with a longer hospital stay than SP-EPRP (WMD: 7.88, 95% CI 0.65, 15.10; *p* = 0.03) (Fig. 2B).

**Fig. 3:**
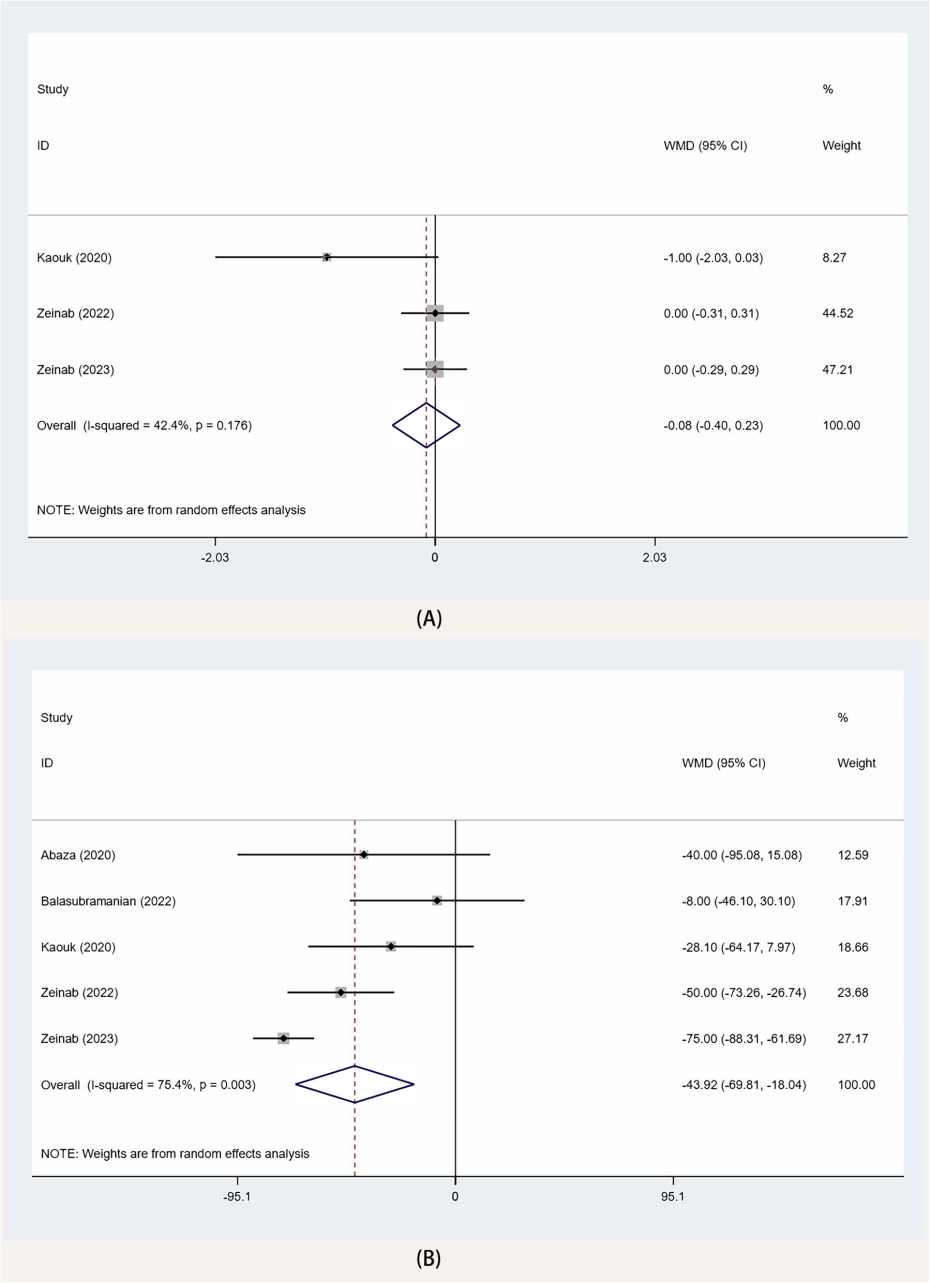
Forest plots of pain scale at discharge and blood loss for SP-TPRP vs SP-EPRP. (**A** pain scale at discharge, **B** blood loss). WMD weighted mean difference, CI = confidence interval

The meta-analysis of 3 studies [10, 14, 15] showed no significant difference between the two groups in postoperative pain scores (WMD − 0.08, 95% CI − 0.40, 0.23; *p* = 0.6) (Fig. 3A).

The meta-analysis of 5 studies [10, 13–16] showed that SP-TPRP was associated with a lower intraoperative blood loss than SP-EPRP (WMD − 43.92, 95% CI − 69.81, − 18.04; *p* = 0.001) (Fig. 3B).

**Fig. 4:**
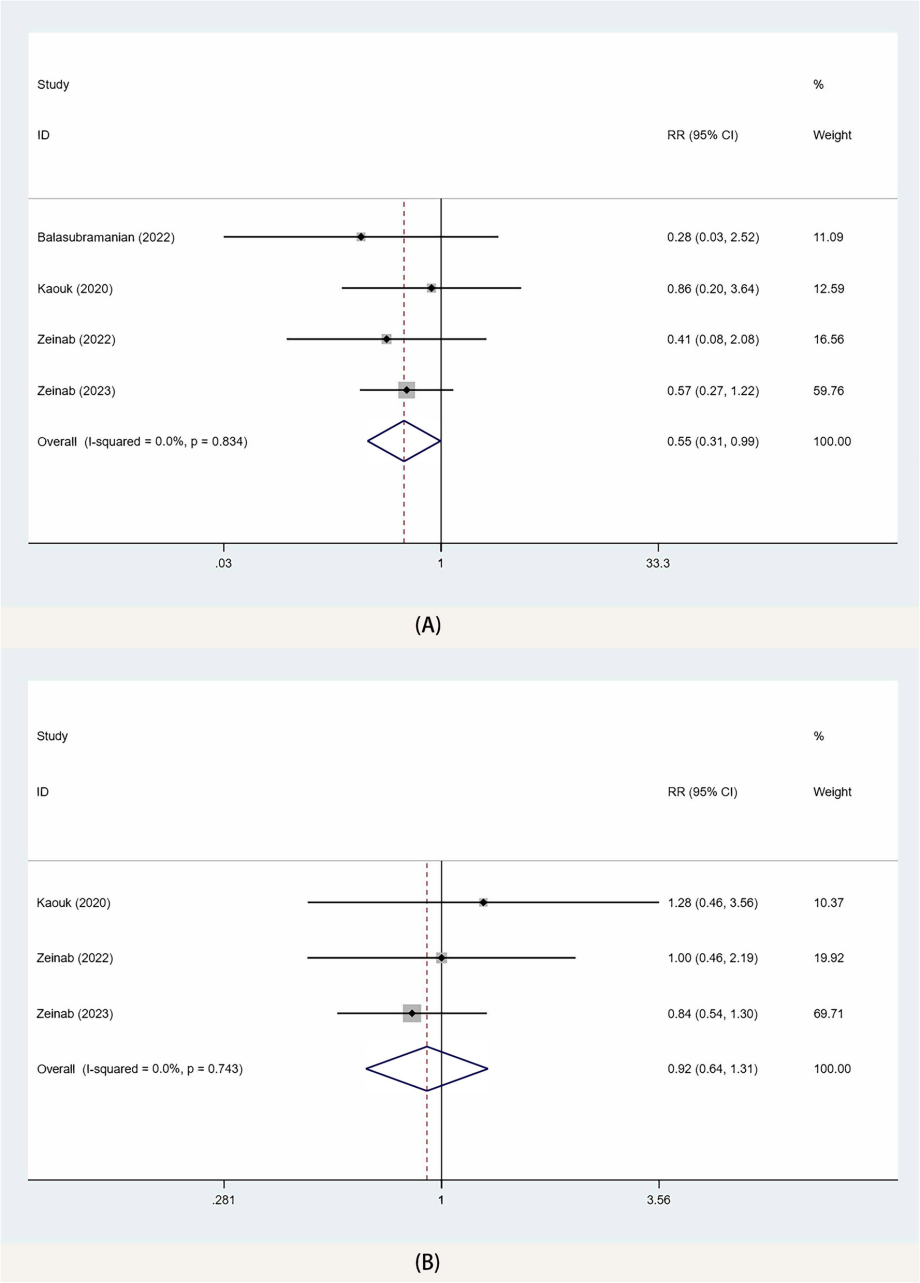
Forest plots of complication for SP-TPRP vs SP-EPRP. (**A** cd-II or greater complications, **B** overall complications). CI confidence interval, RR risk ratio

The meta-analysis of 4 studies [10, 14–16] showed that SP-TPRP was associated with a lower incidence of postoperative Clavien-Dindo II and above complications than SP-EPRP (RR 0.55, 95% CI 0.31, 0.99; *p* = 0.04) (Fig. 4A), but there was no significant difference in the incidence of total postoperative complications between the two groups (RR 0.92, 95% CI 0.64, 1.31; *p* = 0.74) (Fig. 4B).

Furthermore, sensitivity analysis results demonstrated no change in the results for the above perioperative outcomes when we removed each study in turn, suggesting that our results were not influenced by any one study.

### Functional outcomes

**Fig. 5:**
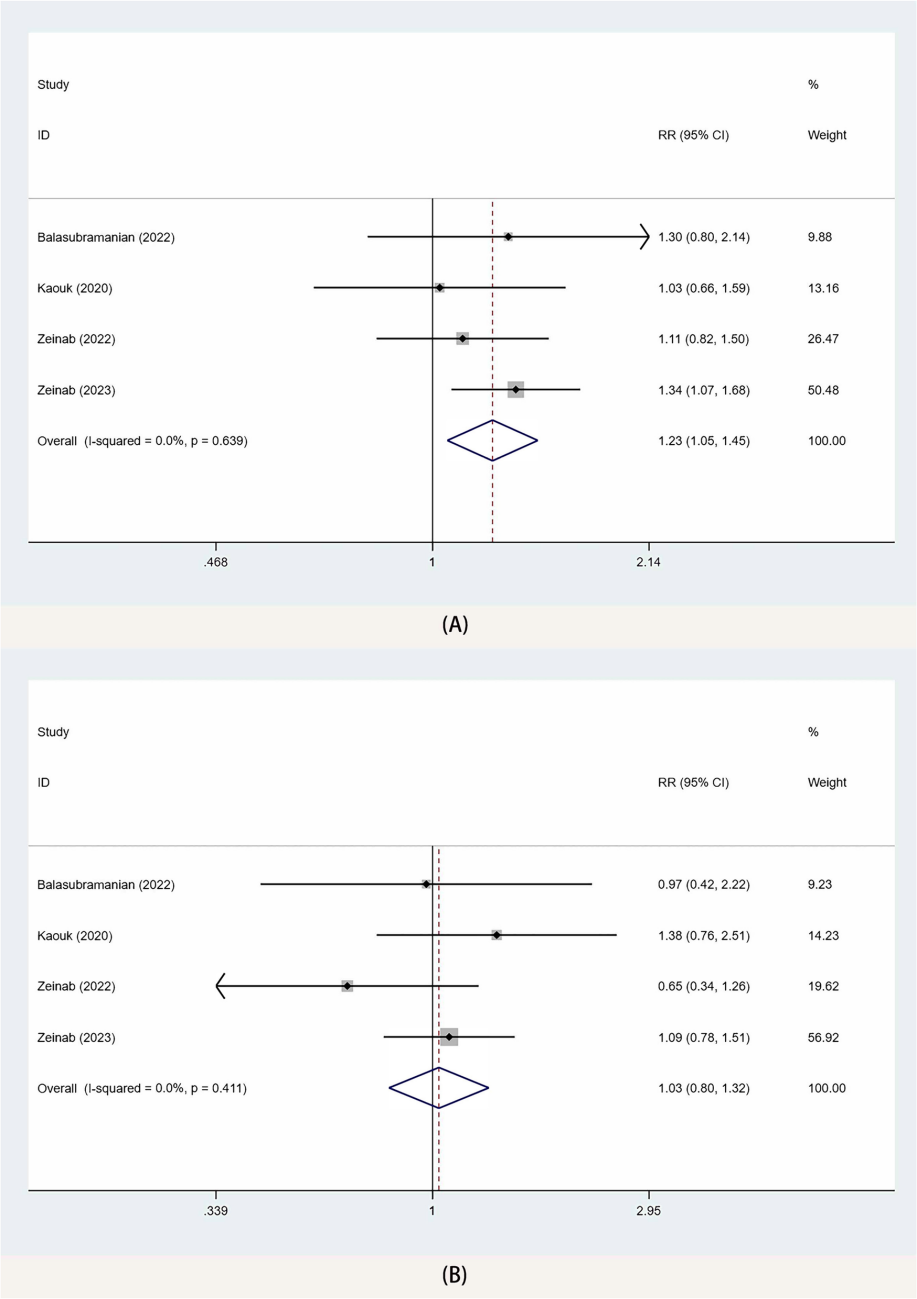
Forest plots of PSM for SP-TPRP vs SP-EPRP. CI confidence interval, RR risk ratio, PSM positive surgical margins

The meta-analysis of 4 studies [10, 14–16] showed that the continence recovery in the SP-TPRP group was better than that in the SP-EPRP group 90 days after the operation (RR 1.23, 95% CI 1.05, 1.45; *p* = 0.04) (Fig. 5A). Moreover, the results of the sensitivity analysis showed no change in the functional outcomes when we removed each study in turn, suggesting that our results were not influenced by any one study.

### Positive surgical margin

The meta-analysis of 4 studies [10, 14–16] showed no significant difference between the two groups in PSM (RR 1.03, 95% CI 0.80, 1.32; *p* = 0.8) (Fig. 5B). Furthermore, the sensitivity analysis reiterated no change in the PSM when we removed each study in turn, suggesting that our results were not influenced by any one study.

### Heterogeneity

Among the seven outcome indicators analyzed in this study, four indicators had moderate or low heterogeneity. However, the hospital stays (*I*^2^ = 94.0%, *p* = 0.03), operation time (*I*^2^ = 96.2%, *p* = 0.868), and blood loss (*I*^2^ = 75.4%, *p* = 0.001) were highly heterogeneous. Given the limited number of included studies, we conducted a meta-regression based on publication year and sample size, as outlined. In accordance with the outcomes of the meta-regression analysis, the potential origins of heterogeneity appear to stem from disparities in publication year and the variations in the size of incorporated research cohorts (*P* > 0.05), as shown in Supplementary File 2.

In light of the constrained inclusivity of the available studies, further subdivision analyses at a sub-group level were regrettably deferred.

### Sensitivity analysis and publication bias

Given the considerable heterogeneity observed in certain outcomes (EBL, LOS, and OT), we conducted sensitivity analyses on the target parameters. Through leave-one-out re-calculation of effect sizes, the results demonstrate the robustness of the findings. Assessment using funnel plots revealed no evidence of publication bias (OT, EBL, LOS), further supported by Begg’s regression tests (*P* > 0.05) (Supplementary File 3), ensuring the integrity of our conclusions in light of potential biases.

## Discussion

To our knowledge, this is the first systematic review and meta-analysis comparing extraperitoneal with transperitoneal surgical approaches in SP-RARP for prostate cancer. We found that both extraperitoneal and transperitoneal SP-RARP are safe and effective. SP-TPRP is superior to SP-EPRP in blood loss, the incidence of postoperative Clavien-Dindo grade II and above complications, and continence recovery, but it is accompanied by a longer hospital stay, which is worthy of our in-depth discussion.

In this study, we extracted the perioperative outcome indicators of SP-EPRP and SP-TPRP surgical approaches for prostate cancer, including operation time, hospital stay, postoperative pain score, blood loss, and the incidence of postoperative complications. The results of the meta-analysis showed that there was no significant difference in operation time between SP-EPRP and SP-TPRP. This is different from the results of previous studies. Uy et al. [6] compared the outcomes of extraperitoneal with transperitoneal surgical approaches in multiport RARP and found that EPRP had a shorter operation time than TPRP. Since EPRP can directly reach the prostate tissue without going through the peritoneum and abdominal cavity during the operation, it has an advantage over TPRP in terms of operation time [7, 17, 18]. The following reasons may lead to this difference: First of all, SP-RARP is a relatively new and complicated surgical method, and the surgical experience of doctors may affect the operation time. Among the studies we included, two studies reported that most surgeons were more proficient in SP-TPRP but less experienced in SP-EPRP, which resulted in the surgery time of SP-EPRP being longer than SP-TPRP [10, 13]. In addition, the patient’s BMI is also an important factor affecting the time of operation [13, 16]. Obesity exerts a discernible impact on the surgical duration and physiological parameters during robot-assisted laparoscopic prostatectomy (RALP). Notably, within the context of transperitoneal robot-assisted laparoscopic prostatectomy (TP RALP), individuals presenting with elevated BMI and assuming the Trendelenburg position tend to exhibit appreciably prolonged operative durations, a pattern that manifests conspicuously [19, 20]. The thicker the abdominal wall fat of the patient, the longer it takes the surgeon doctor to separate the abdominal fat and the longer the operation time [21]. If these biases can be controlled, we expect that the operation time for SP-EPRP will be shorter. In this study, our meta-analysis found that SP-TPRP had a longer hospital stay than SP-EPRP. This may be related to the possibility of peritoneal irritation and intestinal obstruction caused by TPRP [22, 23]. During the operation, since EPRP bypasses the intestine, contact with the intestine is greatly reduced, and intestinal recovery is faster, so the patient’s hospital stay is shortened [24, 25]. While the postoperative pain differential remains inconspicuous between the two approaches, recent investigations have unveiled a superior analgesic efficacy of percutaneous transversus abdominis plane (TAP) block for patients undergoing RARP compared to conventional local anesthetic port infiltration [26, 27]. Employing a robotic-assisted transperitoneal route, this innovative technique specifically targets the anterior branches of the intercostal nerves T7–T11, subcostal nerve T12, ilioinguinal nerve, and iliohypogastric nerve at the TAP plane, intricately interwoven within the sensory neural supply of the skin, musculature, and abdominal wall peritoneal dome [28]. This promising advancement holds the potential to confer a heightened postoperative experience for individuals opting for the SP-TPRP approach.

It should be noted that studies [29, 30] have shown that the patient’s previous abdominal surgery history does not usually affect the incidence of complications after RARP. There was no statistically significant difference in the incidence of total complications between SP-TPRP and SP-EPRP, as was also the case in multiport RARP [31]. However, we found that SP-TPRP has a lower incidence of postoperative Clavien-Dindo II and above complications compared to SP-EPRP. Balasubranian et al. [16] and Zeinab et al. [10] pointed out that SP-EPRP has a higher incidence of lymphoid cysts than SP-TPRP. This may be due to the fact that although SP-EPRP does not destroy the intraperitoneal structure, its operating space is limited, and it lacks lymphatic channels to absorb fluid. Keeping the peritoneal space open during operation or during lymph node dissection may help to reduce such complications. In addition, Reddy et al. [32] pointed out that SP-RARP can provide deterministic treatment of lymphoid cysts and reduce the number of days of abdominal drainage while reducing surgical invasion, but the drainage of lymphocele is also related to the experience of the operator. Therefore, SP-RARP has the advantage of being less invasive, and its postoperative complications, if acceptable, will not affect its rising status in the treatment of prostate cancer.

Our meta-analysis showed that the continence recovery in the SP-TPRP group was better than that in the SP-EPRP group 90 days after the operation. This may be due to the fact that SP-TPRP can better protect the urethral sphincter and external urethral sphincter and reduce the incidence of postoperative continence recovery. It should be noted that intraoperative procedures may affect patients’ continence recovery, such as nerve preservation techniques [33, 34]. In addition, the experience of the surgeon (such as inexperience in SP-RARP or EPRP) and the specific conditions of the patient (such as external urethral sphincter or tissue infiltration around the sphincter) may affect the results. The results of the meta-analysis showed that there was no significant difference in PSM between SP-EPRP and SP-TPRP. The results indicate that the SP-RARP of the two surgical approaches is safe and effective. It should be noted that due to the narrow surgical field of view during SP-RARP, the capsule may be accidentally cut open when surgical instruments are used to remove the prostate through the pelvic levator anal muscle, resulting in occasional false-positive edges [35]. In addition, Freedland et al. [36] found that prostate weight was significantly correlated with postoperative PSM. Consequently, future studies can provide further interpretation by better control of relevant variables.

In recent years, the literature has featured reports examining the feasibility and safety of the SP RARP technique [37]. In comparison to the conventional MP RARP, SP RARP has demonstrated a reduction in superfluous surgical incisions, potentially affording patients an improved postoperative experience with fewer complications [38]. Consequently, a comparative analysis between these two surgical modalities has become indispensably warranted.

In 2021, Fahmy et al. [39] undertook a comprehensive meta-analysis, revealing that SP RARP may potentially lead to shortened hospitalization periods and diminished postoperative pain. However, there were no discernible differences between the two approaches concerning operative time, blood loss, and similar indicators. The validity of these findings was, however, constrained by uncontrollable biases inherent in the included studies. Therefore, for a more conclusive assessment of the comparison between SP RARP and MP RARP techniques in the future, a well-designed randomized controlled trial and long-term follow-up will remain imperative.

At present, SP-RARP is a developing technology, but there are still some problems, such as technical difficulties, lack of experience, and limited available data [40]. The choice of SP-EPRP and SP-TPRP should take into account the experience of the surgeon and the specific conditions of the patient. For experienced surgeons, there is no absolute difference between the two methods. For patients, for patients with low BMI, medium, or small prostate tumors, Moschovas et al. [41] recommend SP-RARP. This is because the patient’s BMI and prostate volume will affect the grasping strength and range of motion of the robotic arm during SP.

### Limitations

Several limitations warrant acknowledgment. First of all, the studies we included are medium-quality retrospective studies with a small sample size, which may lead to the risk of selection bias and reduce the credibility of the results. While the entirety of the investigations was conducted within the ambit of the USA, several of our study metrics yet attain noteworthy levels. Adhering to plausible confounding variables, we conducted a meta-regression analysis; however, due to the constraints imposed by the number of studies included, our capacity to undertake further granularity through sub-group analyses remains circumscribed. We hope that more researchers will pay attention to this topic in the future. Second, even if we use modern statistical methods, some problems still cannot be solved, such as the experience of the surgeons that may interfere with surgical outcomes, nerve-preserving procedures, selection of patient surgical approaches, and other immeasurable confounding factors. Third, most of the studies we included have insufficient median follow-up time, which may lead to a one-sided analysis and lack of comparison of long-term postoperative results between the two groups.

## Conclusions

This study demonstrates that both extraperitoneal and extraperitoneal SP-RARP approaches are safe and effective. Notably, SP-TPRP is superior to SP-EPRP in postoperative blood loss, the incidence of postoperative Clavien-Dindo grade II and above complications, and postoperative continence recovery, but it is accompanied by longer hospital stays. Our findings supplement the comparative data of the two surgical methods, but there are few high-quality studies at present, and this result needs to be further verified by follow-up studies.

### Supplementary Information

**Additional file 1: Table S1.** The demographics of the studies.

**Additional file 2. **The potential origins of heterogeneity appear to stem from disparities in publication year and the variations in the size.

**Additional file 3. **Assessment using funnel plots revealed no evidence of publication bias.

## Data Availability

The data that supports the findings of this study is available from the corresponding authors upon reasonable requests.
