# Supplementary material for: Perioperative, function, and positive surgical margin in extraperitoneal versus transperitoneal single port robot-assisted radical prostatectomy: a systematic review and meta-analysis
Source: World J Surg Oncol. 2023 Dec 12;21:383. doi: 10.1186/s12957-023-03272-7 (PMC10714462; doi:10.1186/s12957-023-03272-7)
Supplement: Supplementary file 1 — Additional file 1: Table S1. The demographics of the studies. [file 12957_2023_3272_MOESM1_ESM.docx]

| The demographics of the studies | | | | |
| --- | --- | --- | --- | --- |
| Variable | No. of studies with available data | WMD/OR | 95% CI | p value |
| Age (years) | 4 | -1.003 | (-1.926,-0.081) | 0.957 |
| BMI (kg/m^2^) | 4 | -0.23 | (-1.42,0.96) | 0.054 |
| PSA, ng/ml | 4 | 0.16 | (-0.59,0.92) | 0.139 |
| Previous  abdominal  surgery, n(%) | 4 | 0.535 | (0.127,2.25) | 0.394 |
| Prostate  volume, mL | 3 | 0.81 | (-1.31,2.93) | 0.458 |
| Biopsy Grade Group | 3 | 0.995 | (0.811,1.227) | 0.995 |
