# Supplementary figures and images for: Perioperative, function, and positive surgical margin in extraperitoneal versus transperitoneal single port robot-assisted radical prostatectomy: a systematic review and meta-analysis
Source: World J Surg Oncol. 2023 Dec 12;21:383. doi: 10.1186/s12957-023-03272-7 (PMC10714462; doi:10.1186/s12957-023-03272-7)

Blood loss


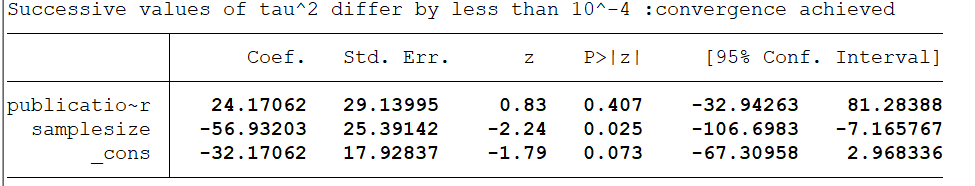


Operate time


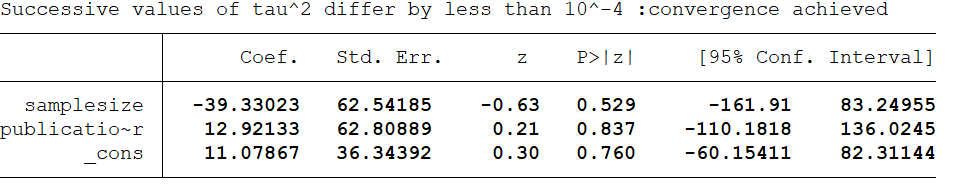


Hospital stay


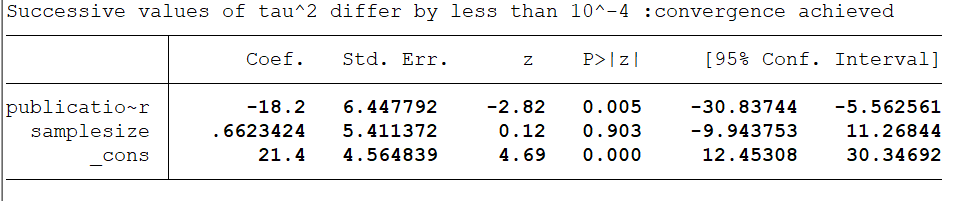

Supplement: Supplementary file 2 — Additional file 2. The potential origins of heterogeneity appear to stem from disparities in publication year and the variations in the size. [file 12957_2023_3272_MOESM2_ESM.docx]

Blood loss


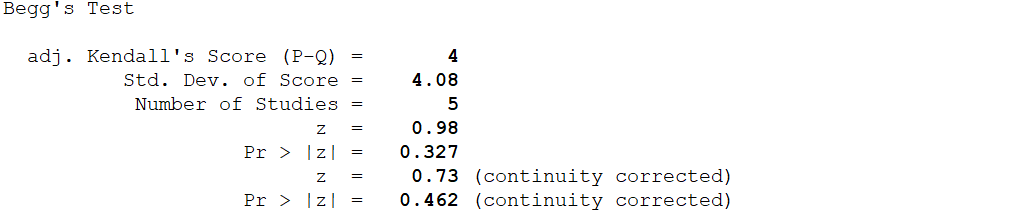


Operate time


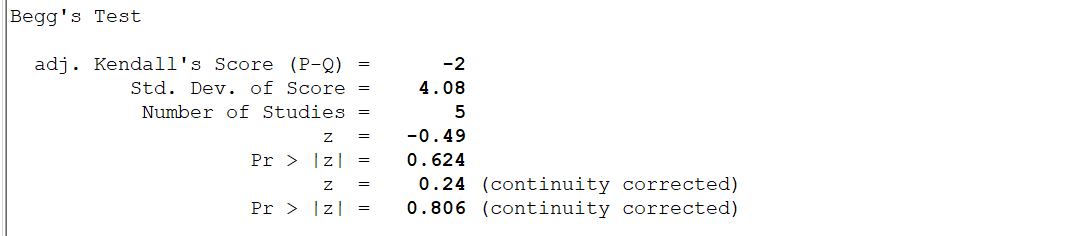


Hospital stay


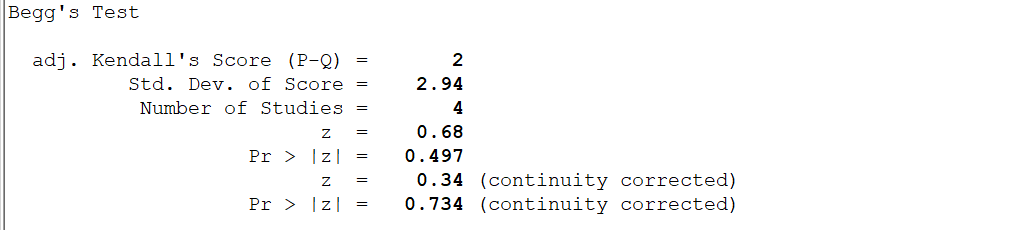

Supplement: Supplementary file 3 — Additional file 3. Assessment using funnel plots revealed no evidence of publication bias. [file 12957_2023_3272_MOESM3_ESM.docx]
